# MetaLP: An integrative linear programming method for protein inference in metaproteomics

**DOI:** 10.1371/journal.pcbi.1010603

**Published:** 2022-10-21

**Authors:** Shichao Feng, Hong-Long Ji, Huan Wang, Bailu Zhang, Ryan Sterzenbach, Chongle Pan, Xuan Guo

**Affiliations:** 1 Department of Computer Science and Engineering, University of North Texas, Denton, Texas, United States of America; 2 Department of Cellular and Molecular Biology, University of Texas at Tyler, Tyler, Texas, United States of America; 3 Texas Lung Injury Institute, University of Texas at Tyler, Tyler, Texas, United States of America; 4 College of Informatics, Huazhong Agricultural University, Wuhan, Hubei, CHINA; 5 Department of Biomedical Engineering, University of North Texas, Denton, Texas, United States of America; 6 School of Computer Science, University of Oklahoma, Norman, Oklahoma, United States of America; University of Richmond, UNITED STATES

## Abstract

Metaproteomics based on high-throughput tandem mass spectrometry (MS/MS) plays a crucial role in characterizing microbiome functions. The acquired MS/MS data is searched against a protein sequence database to identify peptides, which are then used to infer a list of proteins present in a metaproteome sample. While the problem of protein inference has been well-studied for proteomics of single organisms, it remains a major challenge for metaproteomics of complex microbial communities because of the large number of degenerate peptides shared among homologous proteins in different organisms. This challenge calls for improved discrimination of true protein identifications from false protein identifications given a set of unique and degenerate peptides identified in metaproteomics. MetaLP was developed here for protein inference in metaproteomics using an integrative linear programming method. Taxonomic abundance information extracted from metagenomics shotgun sequencing or 16s rRNA gene amplicon sequencing, was incorporated as prior information in MetaLP. Benchmarking with mock, human gut, soil, and marine microbial communities demonstrated significantly higher numbers of protein identifications by MetaLP than ProteinLP, PeptideProphet, DeepPep, PIPQ, and Sipros Ensemble. In conclusion, MetaLP could substantially improve protein inference for complex metaproteomes by incorporating taxonomic abundance information in a linear programming model.

## Author summary

Inferring a reliable list of proteins from identified peptides in metaproteomics is non-trivial because of the prevalence of degenerate peptides in many metaproteome databases. Degenerate peptides are shared among multiple proteins and, therefore, cannot be uniquely attributed to any protein. Here, we developed a protein inference algorithm, MetaLP, for shotgun proteomics analysis of microbial communities to better handle degenerate peptides. Two key innovations in MetaLP were the use of taxonomic abundances as prior information and the formulation of protein inference as a linear programming problem. These features enabled MetaLP to produce substantially more protein identifications in complex metaproteomic datasets than many existing protein inference algorithms.

This is a *PLOS Computational Biology* Methods paper.

## 1 Introduction

Nearly all natural and human-associated ecosystems host microbial communities that are responsible for cycling necessary nutrients [1–4]. Most studies of these microbial communities are based on their metagenomes [5]. But metagenomics results cannot determine whether the genetic potential encoded in the metagenomes is actively expressed as proteins [6], which is a prerequisite for metabolic activities [7]. Metaproteomics identifies proteins in environmental samples of a microbial community and provides insights into their functional states in different conditions. Discovery metaproteomics studies are generally based on high-throughput liquid chromatography-tandem mass spectrometry (LC-MS/MS). Proteins are digested into peptides and analyzed by LC-MS/MS to generate MS/MS data [8]. Identification of proteins from MS/MS data involves two key computational tasks: peptide identification and protein inference. In peptide identification, MS/MS data is searched against a pre-defined protein sequence database to identify a set of peptide-spectrum matches (PSM) and peptides. In protein inference, the identified peptides are assembled into a list of identified proteins.

Inferring reliable proteins from identified peptides is non-trivial because of degenerate peptides that are shared among multiple proteins and, therefore, cannot be uniquely attributed to any proteins. Many methods have been developed to assign unique and degenerate peptides to proteins and rank protein candidates based on their identification confidence. Generally, the protein inference tools are based on either statistical models or machine (deep) learning models. The methods using statistical models embody a set of statistical assumptions concerning the generation of proteins and peptides. For instance, ProteinProphet [9] employed an expectation-maximization algorithm to estimate the probability for a protein to be present in a sample. ProteinLP [10] constructed an optimization problem to handle degenerate peptides and used linear programming to estimate the protein probabilities. MSBayesPro [11] and Fido [12] estimated protein probabilities using Bayesian models. Serang et al. proposed a Bayesian method for computing posterior protein probabilities [13]. EPIFANY [14] used Bayesian networks to infer the protein probabilities. Proteins can also be inferred using machine learning techniques with few or no assumptions [15]. For example, BagReg [16] used a bagging-like strategy with a logistic regression classifier to extract features and score the proteins. DeepPep [17] applied a convolutional neural network to score the protein. However, it is still a major challenge for the existing algorithms to achieve good protein inference in metaproteomics because many peptides are shared among homologous proteins in different microbial species in a complex microbial community. These degenerate peptides are difficult to be assigned to their originating proteins without supplementary biological information.

Protein inference in proteomics has been assisted using other biological information, including transcriptomics [18], functional association network [19]. protein interaction networks [20, 21]. These studies showed that the number of proteins identified in proteomics analysis can be improved by utilizing these types of supplementary information.

In this study, we developed a protein inference algorithm, called MetaLP, for shotgun proteomics analysis of microbial communities. It was optimized for metaproteomics to improve the use of degenerate peptides for protein inference. MetaLP integrated taxonomic abundances as prior information and formulated protein inference as a linear programming problem. These features enabled MetaLP to produce substantially more protein identifications in complex metaproteomics datasets than the existing protein inference algorithms benchmarked here.

## 2 Methods

**Fig 1:**
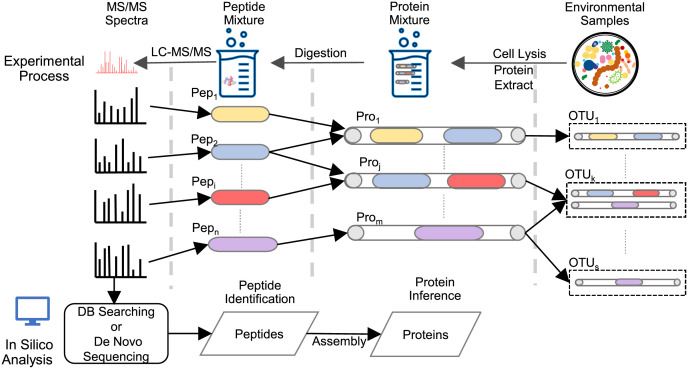
An example of protein inference tripartite graph given identified peptides and protein annotations.

Since a degenerate peptide can be mapped to multiple proteins and a protein can be generated by more than one organism, the input can be represented as a tripartite graph (Fig 1. The left is a set of identified peptides. The middle is a set of candidate proteins that have at least one constituent peptide. And the right is a set of species that may produce those proteins. The ability to detect a protein present in the samples depends on how we assign the unique and degenerate peptides to the proteins that have truly generated them and the abundance of that protein in the samples. We assume that a species with a larger population size generates more proteins in a metaproteome sample. Because this assumption may not be strictly correct in many real-world communities, we relax the taxonomic level to operational taxonomic unit clusters, each of which may contain multiple organisms that have similar abundance. We formulate the protein inference as an optimization problem for finding a likely smallest subset of candidate proteins that best ‘explain’ both the identified peptides and the operational taxonomic unit clusters with known abundances. To solve the optimization problem, we designed a linear programming (LP) model, named MetaLP, to incorporate the peptide identification results and taxonomic cluster abundances from metagenomics sequencing. The contribution allocation of degenerated peptides can be abstracted as an optimization problem as shown in the constraints in MetaLP. And linear programming is well known for finding the optimal means of allocating finite resources among competing entities. In addition, it is easy and convenient to incorporate the species abundance information in our proposed model. Here, we expressed the joint probability with a chain rule to transform it into a chain of conditional probabilities, which could be easily added as logical constraints. All the above factors inspired us to investigate the linear programming method for the protein inference problem.

The LP model can be solved quickly by existing LP solvers [22]. The following sections will describe the MetaLP model and the notations used in it, and explain the workflow of protein inference based on MetaLP model and the estimation of the abundance of operational taxonomic unit (OTU) clusters. Here, OTU is defined as an operational unit used to classify groups of closely related organisms at the genome level. MetaLP is freely available under the GNU GPL license at https://github.com/Biocomputing-Research-Group/metaLP, where step-by-step installation and usage were provided.

### 2.1 Notations

Suppose we have *n* peptides identified by an existing database searching tool, *m* candidate proteins containing these identified peptides, and *s* candidate operational taxonomic unit clusters. We use *pep*_*i*_, *pro*_*j*_, and *otu*_*k*_ to denote the presences of *i*th peptide, *j*th protein, and *k*th operational taxonomic unit cluster measured in the metaproteome samples, respectively.

Let *MS* denote the observed mass spectra data. *P*(*pep*_*i*_|*MS*) is the probability that the *i*th identified peptide exists and is measured in the metaproteome samples. *P*(*pro*_*j*_|*MS*) denotes the probability that the *j*th protein exists and is measured in the metaproteome samples. *P*(*otu*_*k*_|*MS*) denotes the probability that the *k*th operational taxonomic unit cluster exists and is measured in the metaproteome samples, which serves as priors to adjust *P*(*pep*_*i*_|*MS*).

### 2.2 MetaLP model

A peptide is present if at least one of its parent proteins and one of its operational taxonomic units are present, which can be described as in Eq 1.

$$\begin{array}{c} P\left(pep_{i}|MS\right)=\sum_{j=1}^{m}\sum_{k=1}^{s}P\left(pep_{i},pro_{j}|otu_{k},MS\right)P\left(otu_{k}|MS\right) \end{array}$$ (1)

If the *j*th protein and the *k*th operational taxonomic unit cluster do not contain the *i*th peptide, the corresponding probability has the value of zero. Here, the joint probability, *P*(*pep*_*i*_, *pro*_*j*_|*otu*_*k*_, *MS*), denotes the probability that the *i*th peptide and the *j*th protein are present and measured, given that the *k*th operational taxonomic unit cluster. This joint probability relates the peptide probability to the protein probability given the presence of operational taxonomic units.

We formulate the protein inference problem as an optimization problem using the linear programming model. The objective is to minimize $\sum_{j=1}^{m}P\left(pro_{j}|MS\right)$ so as to shrink some protein probabilities to 0.

$$\begin{array}{c} minimize\sum_{j=1}^{m}P\left(pro_{j}|MS\right) \end{array}$$ (2)

The MetaLP model has the following three types of constraints.

$$\begin{array}{c} \forall i,P\left(pep_{i}|MS\right)+\epsilon \geq \sum_{j=1}^{m}\sum_{k=1}^{s}P\left(pep_{i},pro_{j}|otu_{k},MS\right)P\left(otu_{k}|MS\right) \end{array}$$ (3)

$$\begin{array}{c} \forall i,P\left(pep_{i}|MS\right)-\epsilon \leq \sum_{j=1}^{m}\sum_{k=1}^{s}P\left(pep_{i},pro_{j}|otu_{k},MS\right)P\left(otu_{k}|MS\right) \end{array}$$ (4)

$$\begin{array}{c} \forall i,\forall j,P\left(pro_{j}|MS\right)\geq \sum_{k=1}^{s}P\left(pep_{i},pro_{j}|otu_{k},MS\right)P\left(otu_{k}|MS\right) \end{array}$$ (5)

The constraints 3 and 4 control the difference between the probability of a peptide being measured by LC-MS/MS and the probability of it being identified. The *ϵ* denotes the difference between the observed and theoretical peptide probabilities. This parameter reflects how confident the peptide identification tool is. For example, *ϵ* = 0 means that the input peptide probability is perfectly accurate. In our experiments, we used 0 as the default setting. The constraint 5 is used to find the minimum value in *P*(*pro*_*j*_|*MS*). Since only a subset of candidate proteins are truly present and measured in the samples, some protein probability values should be zero. Thus, we minimize $\sum_{j=1}^{m}P\left(pro_{j}|MS\right)$. To achieve this objective function, the LP solver needs to adjust the joint probability, *P*(*pep*_*i*_, *pro*_*j*_|*otu*_*k*_, *MS*), based on constraint 5 to set some protein probabilities to zero. The MetaLP model can be quickly solved with standard LP solvers. In this study, we used Gurobi Optimizer v9.1.2 [23].

### 2.3 OTU probability estimation

OTU is considered as the operational unit to classify groups of closely related organisms. In this work, we assume that a microorganism with a larger population may generate more proteins. Thus, the OTU population serves as the prior probability that a protein originates from the OTU. We defined the OTU clusters based on either the metagenomic binning or the 16S rRNA sequence clustering. Specifically, the OTU clusters were constructed in one of the following three ways depending on the input data. When the reference genomes were available for the microbial community, the DNA sequencing reads were mapped to the reference genomes using BBSplit in BBTools package version 38.94 [24]. When the microbial genomes need to be reconstructed from the shotgun sequencing data of the metagenome samples, the DNA reads were assembled using metaSPAdes [25] and metagenome-assembled genomes were binned using MetaBAT2 [26] from MetaWRAP [27]. The DNA reads were then mapped to all the bins using Bowtie2 [28], and the abundance of each bin was computed as the number of DNA reads in that bin. Here, the bins and clusters are used interchangeably. When the 16s rRNA gene sequencing data was provided, sequences were clustered into bins based upon similarity using vsearch version 11 [29]. The result OTU clusters were annotated by searching against the RDP database [30], and the corresponding results were used to locate reference genomes and build the matched protein databases. All the above tools were used with their default parameters.

Once the OTU clusters were constructed, the protein sequences can be predicted from reference genomes or assembled genomes and assigned to the corresponding clusters using the mapping between genomes and clusters. Here, we do not need the OTU clusters at high granularity levels. It would be impractical to have each cluster contain the sequences only from one species since existing reference genomes may not match the organisms in the experimental samples. Also, the results in Section 3 demonstrate that the OTU clusters generated by MetaBAT2 were accurate enough to significantly improve the number of identified proteins. The probability of the OTU clusters was calculated as in Eq 6.

$$\begin{array}{c} P\left(otu_{k}\right)=\frac{\#Reads\in OTU\;Cluster_{k}}{\#Total\;Reads} \end{array}$$ (6)

In Eq 6, each OTU cluster may contain multiple organisms. Given that the mass spectra are generated independent of the metagenome sequencing data, we have *P*(*otu*_*k*_) = *P*(*otu*_*k*_|*MS*). We also used the sequencing depth to estimate the OTU probability. The probability of the OTU clusters was calculated as in Eq. A in S1 Text. The Benchmarking of protein identification at 1% FDR were shown in Tables F and G in S1 Text. MetaLP achieved the best performance compared to the other tools, including two other variants of MetaLP.

### 2.4 Schematic overview and implementation

**Fig 2:**
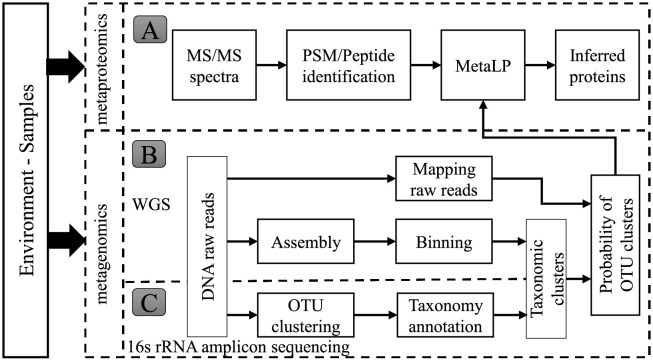
The schematic overview of MetaLP.

The schematic overview of our MetaLP is shown in Fig 2, which includes the metaproteomics pipeline (Part A) and metagenomics pipeline (Parts B & C). In our experiments, the input data for both pipelines were measured from the same biological replicates to ensure that the protein databases matched with the mass spectra data.

In the metaproteomics pipeline, the mass spectra data were extracted from raw data and reformatted by MSConverter [31]. MetaLP requires a list of PSMs or peptides with probability scores generated by database search engines or filters. A sample input of PSMs was provided in our GitHub repository. We tested with two database search engines, i.e., Comet [32] and Sipros-Ensemble [33, 34]. PeptideProphet [35] was used as a filter to re-rank PSMs and produce peptide candidates with probabilities, i.e., *P*(*pep*|*MS*).

In the metagenomics pipeline, we implemented two workflows to obtain OTU clusters, one based on the whole-genome DNA sequencing (WGS) (Part B) and the other based on 16s rRNA gene sequencing data (Part C). Details about these two workflows are described in Section 2.3. The resultant probabilities of OTU clusters were used in our MetaLP model.

## 3 Experiments and results

### 3.1 Experimental design and benchmark datasets

MetaLP was compared with five other popular protein inference algorithms, including ProteinLP [10], ProteinProphet [9], Sipros-Ensemble [33], PIPQ [36] and DeepPep [17]. To investigate the importance of OTU priors in MetaLP, we also implemented a variant of MetaLP, denoted as MetaLP*, without the probabilities of OTU clusters (i.e., all *P*(*otu*|*MS*) were set to 1). Note that Sipros-Ensemble is a complete framework that contains database searching, filtering, and protein inference, which are denoted as SE-S, SE-F, and SE-PI, respectively, in the following sections. For the PIPQ algorithm, we employed all the options provided, which include equal division, multiple counting, and linear programming, and the results from the variants of PIPQ are labelled as PIPQ-e, PIPQ-m, and PIPQ-lp. We used two combinations of database search engines and filters, one with Comet [32] as a search engine and PeptideProphet [35] as the filtering algorithm and the other with Sipros-Ensemble [33] for both database searching and filtering.

The performance of MetaLP was evaluated on four microbial communities, including three metaproteome datasets from mock communities [37], three metaproteome datasets from marine communities [38], three metaproteome datasets from soil communities [39], and one human gut metaproteome dataset [40]. All metaproteome samples were measured on the LTQ Orbitrap Elite mass spectrometers (Thermo Scientific) using the Multidimensional Protein Identification Technology (MudPIT) approach [41].

For the mock community, the taxonomy of the bacteria was known, so the genomes of corresponding bacteria were used as OTU clusters. For the marine and soil metaproteome samples, the organism compositions were unknown and the metagenomic sequencing data was used to construct OTU clusters. For the human gut metaproteome, we used 16s rRNA gene sequencing data to obtain OTU clusters. Table 1 shows the number of OTU clusters from each metaproteome and the percentages of the cross-cluster peptides which was defined as the peptides shared across more than one OTU cluster. The higher the percentage of cross-cluster peptides was, the more closely related species were present in the metaproteome samples. The experimental design was based on two factors: the complexity of microbial community composition and the estimation accuracy of OTU clusters. As shown in Table 1, the mock community, and human gut data have relatively lower complexity and the marine metaproteomes have relatively higher complexity. We have three routes to estimate the OTU probabilities. The route using known species was considered to have high OTU estimation accuracy. The route with unknown species using WGS data has low OTU estimation accuracy, and the route using 16s rRNA data is in the middle. We want to investigate the performance of MetaLP under different levels of microbial composition complexities and OTU estimation accuracy. From the experimental results, we found that MetaLP could achieve greater improvement for more complex microbial communities and was not sensitive to the OTU estimation accuracy.

**Table 1:** The number of taxonomic clusters and percentage of cross-cluster peptides of all the identified peptides for different microbial communities.

Metaproteomes^a^	P2	P3	P4	M1	M2	M3	S1	S2	S3	HG
# OTU clusters	22	22	22	169	169	169	71	53	80	13
% Cross-cluster peptides	17%	18%	18%	34%	35%	34%	21%	16%	20%	19%

^a^ Metaproteomes: three mock microbial communities: P2, P3, P4; three marine microbial communities: M1, M2, M3; three soil microbial communities: S1, S2, S3; a human gut metaproteome: HG

### 3.2 Algorithm testing

Benchmarking datasets were searched using Comet 2018.01 rev. 2 and Sipros-Ensemble Version 1.2. The precursor mass tolerance was set to 0.09 Da, fragment mass tolerance was set to 0.01 Da, peptide mass range was set from 700 Da to 7000 Da, Trypsin/P was used for digest enzyme, and the allowed number of missing cleavage was set to three. All other parameters were set to default. Comet and Sipros-Ensemble were executed on a workstation with two 2.4GHz Intel Xeon E5–2680 v4 CPUs and 64 GB memory. The PSM identification results were filtered by PeptideProphet from TPP v5.2.0 with default configuration settings and executed on a workstation with one 2.6 GHz Intel(R) Xeon(R) Silver 4112 CPU and 32 GB memory. The protein inference tools were executed on a workstation with a 2.6 GHz Intel(R) Xeon(R) Silver 4112 CPU and 32 GB memory. The metagenomic sequencing data were assembled and binned by metaSPAdes and MetaBAT2, respectively, using the default parameters on a workstation with an Intel Xeon E5–4640 CPU and 512 GB memory. The 16s rRNA gene sequencing data were clustered and annotated by vsearch v11 on a workstation with a 2.3 GHz Intel(R) Xeon(R) Gold 5118 CPU and 32 GB memory.

The time and memory consumption of MetaLP is shown in Table D in S1 Text. We picked one dataset from each microbial community. MetaLP could finish the protein inference in less than a minute. The memory usage of MetaLP is related to the number of peptides, and it used 2.5 GB memory for the marine data set with 25,411 peptides. We believe that MetaLP can be easily running at a regular workstation without any memory issues.

### 3.3 Evaluation

For all the benchmark methods and datasets, we applied the target-decoy strategy [42] to control the false discovery rates (FDR) at the PSM, peptide, and protein levels. The decoy proteins were generated by reversing the target protein sequences. The FDR is estimated as in Eq 7. The identified peptides with FDR controlled at 1% were used as input for MetaLP and other benchmarked tools. For MetaLP, the probabilities of OTU clusters for decoy proteins were set to the same values as for the corresponding target proteins. We evaluated the performance of all methods by the number of target proteins/protein groups with the protein level FDR controlled at 1%. We used protein groups when identified peptides were not distinguishable. Protein groups were defined as the set of proteins with the same set of identified peptides, which are not distinguishable. To avoid double standards for evaluation, we applied the same rule to define protein groups for all the benchmarked algorithms, i.e., at least one unique peptide needed for an identified protein/protein group.

In addition to the commonly used targetdecoy strategy in Eq 7, we also use the “picked” target-decoy strategy [43] to control the FDRs. Due to the homology between proteins and species, Eq 7 may not work well at protein-level. The “picked” target-decoy strategy may address this problem by counting the proteins from the same gene once and keeping the one with the highest score for FDR estimation in a pair of target and decoy proteins.

$$\begin{array}{c} FDR=\frac{\#Decoys}{\#Targets} \end{array}$$ (7)

### 3.4 Performance comparison on mock communities

The MetaLP was compared with eight different combinations of existing database searching and filtering algorithms on the three mock community samples (Table 2 and Fig A in S1 Text). Table 2 shows the identifications of proteins filtered at 1% FDR. Across the three mock metaproteomes, MetaLP generated more protein identifications than any other protein inference algorithm. It achieved a 2.1% to 4.7% increase in the number of identified proteins compared to the second-best among the benchmarked algorithms. Without considering MetaLP, all the other benchmarked tools performed similarly on the mock communities. The MetaLP without using OTU cluster probabilities could produce comparable results to the second-best method. Fig A in S1 Text shows the overlap of identified proteins among benchmarked approaches. On average, 320 proteins were uniquely identified by MetaLP, which is the second-best among all the benchmark methods. DeepPep had the most number of uniquely identified proteins, but it obtained fewer proteins in total. The results for the mock community filtered by the picked target-decoy strategy at 1% FDR are shown in Table A in S1 Text. MetaLP also achieves a considerable improvement of identified proteins (2.6% to 5.3%). The identified proteins with varied FDRs are shown in Figs J and N in S1 Text, which demonstrate that MetaLP outperformed other benchmarked protein inference algorithms.

**Table 2:** Benchmarking of protein identification at 1% FDR using three mock metaproteomes and a human gut metaproteome.

	Database search engines & Filters							
	Comet with PeptideProphet				SE-SF^c^			
Metaproteomes^a^	P2	P3	P4	HG	P2	P3	P4	HG
PI tools^b^								
SE-PI	8627	7524	6605	3602	9153	7258	7006	3393
LP	8684	7605	6686	3945	9168	7324	7038	3682
PP	8678	7620	6579	3895	9157	7327	7010	3571
DP	8641	7617	6452	2762	5330	2758	6916	2370
PIPQ-e^d^	8648	7612	6681	3976	9193	6403	6926	3872
PIPQ-m^d^	8682	7587	6766	4085	8158	7201	6463	3843
PIPQ-lp^d^	8764	7721	6746	4141	9206	6657	6933	2931
MetaLP*	8663	7581	6676	3832	9137	7277	7007	3527
MetaLP	**9032**	**7883**	**6937**	**4233**	**9487**	**7669**	**7335**	**4004**

^a^ Metaproteomes: three mock microbial communities: P2, P3, P4; a human gut metaproteome: HG.

^b^ Protein inference tools(PI tools): SE-PI, Sipros Ensemble protein inference; LP, ProteinLP; PP, ProteinProphet; DP, DeepPep; MetaLP*, MetaLP model without OTU cluster probabilities.

^c^ SE-SF: Sipros-Ensemble searching and filtering.

^d^ PIPQ with three different options, i.e., PIPQ-e (equal division), PIPQ-m (multiple counting), PIPQ-lp (linear programming).

^e^ The best entry was in bold, the second best was underlined.

### 3.5 Performance comparison on human gut community

The MetaLP and benchmarked methods were also compared on a human gut microbial sample with matched MS/MS proteomics data and 16s rRNA gene sequencing data. For MetaLP, the OTU clusters were generated and annotated as in Section 2.3. The protein database was constructed by extracting translated proteins from the NCBI database [44] using the corresponding taxonomy identifier provided by the original study [40]. The identified proteins at 1% FDR are shown in Table 2. Similar to the mock community samples, our MetaLP identified 2.2% more proteins for the Comet & PeptideProphet pipeline and 3.4% more proteins using the Sipros-Ensemble pipeline than the second-best one Fig D in S1 Text demonstrates that 406 proteins were only inferred by MetaLP, which outperformed all the other benchmarked methods. The identified proteins with varied protein level FDRs are shown in the Figs M and Q in S1 Text. MetaLP achieves 6.8% and 7.1% more identified proteins for the Comet & PeptideProphet pipeline and Sipros-Ensemble framework, respectively, with FDR controlled by the picked target-decoy strategy.

### 3.6 Performance comparison on marine and soil communities

We also compared the performance of MetaLP and other tools on marine and soil microbial communities samples. All samples had matched metagenome and metaproteome datasets. The OTU clusters were processed as in Section 2.3. The microbial compositions were highly complex for the marine and soil microbial communities, given the large numbers of OTU clusters and the high percentages of cross-cluster peptides as shown in Table 1. Table 3 shows the identifications of proteins filtered at 1% FDR. In general, the Sipros-Ensemble pipeline produced more identified proteins than the Comet & PeptideProphet pipeline, no matter which protein inference tool was used. Across all the pipelines and the metaproteome samples, MetaLP achieved the highest number of protein identifications. For the Comet & PeptideProphet pipeline, the improvements of MetaLP were 14.1%, 16.7%, and 13.3% compared to the second-best method on the three marine metaproteome data sets. For the Sipros Ensemble pipeline, the improvements of MetaLP were 19.5%, 18%, and 8.4% compared to the second-best method on the three marine metaproteome datasets. For the three soil data sets, MetaLP identified 5%, 4.2%, 4.5%, 7.6%, 7.7%, and 11.9% more proteins compared to the second-best ones for Comet & PeptideProphet and Sipros Ensemble pipelines, respectively. Figs B and C in S1 Text demonstrate that MetaLP uniquely inferred approximately 20,000 proteins for each marine microbial sample and approximately 2,000 proteins for each soil microbial sample, which outperformed all the other benchmarked methods. The identified proteins vs. FDRs are shown in Figs K, L, O, and P in S1 Text using two target-decoy strategies. The results at 1% FDR controlled by the picked target-decoy strategy are shown in Table B in S1 Text. MetaLP obtains 9.1% to 30% more identified proteins for the marine metaproteomes and 3.7% to 12% more identified proteins for the soil metaproteomes. From these experimental results, we found that MetaLP provided a significant improvement of protein inference in the complex microbial communities, such as marine and soil communities, than the simple communities, i.e., the mock communities and the human gut microbiota.

**Table 3:** Benchmarking of protein identification at 1% FDR using three marine metaproteomes and three soil metaproteomes.

	Database search engines & Filters					
	Comet with PeptideProphet			SE-SF		
Marine Metaproteomes^a^	M1	M2	M3	M1	M2	M3
PI tools^b^						
SE-PI	9183	9990	10465	11160	10720	10405
LP	10052	10903	11442	15021	14290	14042
PP	9329	10127	10655	14162	14221	15547
DP	3021	3608	3679	3435	2758	4657
PIPQ-e^d^	7789	8063	8471	14052	10035	15249
PIPQ-m^d^	10027	8833	8954	12894	14136	16470
PIPQ-lp^d^	10412	10986	11775	15532	15441	16619
MetaLP*	9180	9964	10412	13387	12248	11062
MetaLP	**11881**	**12826**	**13342**	**18562**	**18228**	**18030**
Soil Metaproteomes^a^	S1	S2	S3	S1	S2	S3
PI tools^b^						
SE-PI	4901	5272	4906	5859	6216	6005
LP	5021	5373	4935	6546	6464	6520
PP	4941	5378	4946	6528	6512	6632
DP	3115	3037	3427	3640	3369	4307
PIPQ-e^d^	5231	5443	5153	5905	5553	6079
PIPQ-m^d^	5236	5512	5208	6229	6339	6739
PIPQ-lp^d^	5255	5535	5203	5955	5748	6662
MetaLP*	4916	5303	4849	6230	6306	6262
MetaLP	**5516**	**5769**	**5440**	**7042**	**7013**	**7544**

^a^ Three marine and three soil metaproteomes M1 (Marine 1), M2 (Marine 2), M3 (Marine 3), S1 (Soil 1), S2 (Soil 2), S3 (Soil 3).

^b^ Protein inference tools(PI tools): SE-PI, Sipros Ensemble protein inference; LP, ProteinLP; PP, ProteinProphet; DP, DeepPep; MetaLP*, MetaLP model without OTU cluster probabilities.

^c^ SE-SF: Sipros-Ensemble searching and filtering

^d^ PIPQ with three different options, i.e., PIPQ-e (equal division), PIPQ-m (multiple counting), PIPQ-lp (linear programming).

^e^ The best entry was in bold, the second best was underlined.

### 3.7 Performance comparison using a synthetic database with real-world decoys

All the experiments above used reverse sequences as decoys to estimate FDRs. To make the benchmarking a better simulation of real-world analysis, we combined the marine database containing 2,876,135 protein sequences and the human gut database containing 106,140 protein sequences and searched the human gut MS/MS dataset against this synthetic database with reverse decoys. The FDR was estimated as before, but we regarded protein identifications from the marine database as false identifications and protein identifications from the human gut database as true identifications after the FDR was controlled at 1%. Note that protein groups that contained at least one human gut protein were classified as true. The probabilities of the OTUs to which marine proteins belonged were set to zero since we knew no marine proteins were present in the human gut samples. This is one of the key contributions of MetaLP that it can utilize the genomic information, which informed us that no marine microbes were in the proteome samples. The identification results from all benchmarked tools are shown in Table 4. The accuracy is defined as the ratio of the true protein number to the false protein number at 1% FDR. All the methods achieved more than 93% accuracy. MetaLP obtained the highest accuracy and yielded the largest number of true proteins and the fewest false proteins.

**Table 4:** Identification of human gut proteins at 1% FDR from a synthetic database containing both the marine database and the human gut database.

	Total	False^b^	True^c^	Accuracy
PI tools^a^				
SE-PI	3221	69	3152	0.979
LP	3509	76	3433	0.978
PP	3433	72	3361	0.979
DP	2287	51	2236	0.978
PIPQ-e^d^	3582	94	3488	0.974
PIPQ-m^d^	3517	234	3283	0.933
PIPQ-lp^d^	3543	75	3468	0.979
MetaLP*	3372	68	3304	0.980
MetaLP	**3742**	**8**	**3734**	**0.998**

^a^ Protein inference tools(PI tools): SE-PI, Sipros Ensemble protein inference; LP, ProteinLP; PP, ProteinProphet; DP, DeepPep; MetaLP*, MetaLP model without OTU cluster probabilities.

^b^ False IDs of marine proteins.

^c^ True IDs of human gut proteins.

^d^ PIPQ with three different options, i.e., PIPQ-e (equal division), PIPQ-m (multiple counting), PIPQ-lp (linear programming).

^e^ The best entry was in bold, the second best was underlined.

## 4 Discussion

### 4.1 Performance assessment of different OTU estimation strategies

In order to assess the impact of different ways of estimating OTU probabilities, we did a comparison for mock communities and the human gut metaproteome because they have similar microbial complexities (there were 22 species in mock communities and 13 species in the human gut metaproteome). The OTU probability estimations for the mock communities and the human gut metaproteome used the whole genome sequencing (WGS) data and the 16s rRNA sequencing data, respectively. For the mock communities, MetaLP identified 2.6% and 3.9% more proteins on average compared to the second best for the Comet & PeptideProphet pipeline and Sipros-Ensemble framework, respectively. For the human gut dataset, MetaLP identified 2.2% and 3.4% more proteins compared to the second best for the Comet & PeptideProphet pipeline and Sipros-Ensemble framework, respectively. The comparison revealed that the performance improvement using the 16s rRNA data was slightly lower than using the WGS data but not significant. It may be because of the better accuracy of the OTU clustering using the WGS data than the 16s rRNA sequencing data. In general, MetaLP is not sensitive to the OTU estimation accuracy.

### 4.2 Accuracy on proteins containing degenerate peptides

To investigate the performance of different methods in tackling the peptide degeneracy issue, we present the identification results of five methods when inferring proteins containing degenerate peptides (Table E in S1 Text). Since there is no ground truth for the proteins that can be measured by the MS instrument, following the evaluation metric in ProteinLP [10] and set stringent cutoff probabilities to annotate the positives using the results generated by ProteinProphet. For all the datasets, we count the number of true positives and false positives identified by ProteinProphet (PP), ProteinLP (LP), DeepPep (DP), and MetaLP among their top-k ranked proteins. The value of k was set to the number of proteins with high probabilities reported by ProteinProphet. The cutoff probabilities were set to 0.99 for mock community data sets and 0.98 for marine, soil, and the human gut metaproteome data sets. The true positives (TP) and the false positive (FP) proteins were set to the target and decoy proteins in the top-k ranked proteins. We split the identified proteins into two categories: “degenerate proteins” were proteins that shared peptides with other proteins, and “simple proteins” were those that had at least one unique peptide not shared by any other protein.

From Table E in S1 Text, we can reach the following conclusions. First, DeepPep reported the smallest number of TP degenerate proteins and the largest number of FP simple proteins in all datasets. Given that DeepPep was based on a deep convolutional neural network framework to predict the protein set from a proteomics mixture, it may not generalize well to the data sets used in this study. Second, ProteinProphet, ProteinLP, and MetaLP* identified nearly the same numbers of simple and degenerate proteins in most cases. This showed that these methods have similar discrimination power on ranking degenerate and simple proteins. Third, MetaLP was able to identify more TP degenerate proteins and fewer FP proteins than ProteinProphet, ProteinLP, and MetaLP* on all the datasets. We reasoned that the MetaLP model can prioritize some degenerate proteins from others with the extra information from metagenome sequencing data. Therefore, our MetaLP method can handle the degenerate protein issue better than the benchmarked tools.

### 4.3 Investigation of parameter *ϵ* and the quality of identified peptides

The only parameter, *ϵ*, in our MetaLP model was set to zero by default. To investigate the effect of this parameter, we ran MetaLP on the human gut, marine, and soil data sets by adjusting the values of *ϵ* from 0 to 0.9 with the step size of 0.1. Since all the probabilities were less than or equal to 1.0, we left out the parameter value of 1.0. Figs E to H in S1 Text show the numbers of identified proteins under the different values of *ϵ*. The performance differences between the optimum value and the default value of *ϵ* were 2.8%, 5.7%, 4.9%, and 3.9% on average for mock, marine, soil, and human gut microbial communities, respectively. Even though *ϵ* = 0 is not the best choice, the improvement from adjusting *ϵ* is marginal.

To assess the impact of the identified peptide quality on protein inference, we tested MetaLP and benchmarked algorithms using all the reported peptides by the search engines without filtering. The protein inference results at 1% FDR are shown in Table C in S1 Text. The results demonstrate that MetaLP still performed the best among all the benchmarked algorithms, but there was a slight drop in the improvement of MetaLP compared to ProteinLP, i.e., the improvement of MetaLP dropped by 0.9%, 2.1%, and 4.9% on average for mock community datasets, marine metaproteomes, and soil metaproteomes, respectively. Note that we did not test the protein inference function in the Sipros-Ensemble framework because it required the filtered peptides to infer proteins. From Table C in S1 Text, we can find that PSMs/peptides do not need to be highly confident for the benchmarked tools to infer proteins. As long as the probabilities reported by the search engines reflect how likely a peptide matches with a measured spectrum, existing protein inference tools can adjust the protein probabilities properly. The *ϵ* parameter may improve the results when there is a discrepancy between the reported probability and the actual probability of a PSM/peptide.

### 4.4 Analysis of the taxonomy information from protein identification results

To take a deeper look at the identified proteins (only) inferred by MetaLP from the taxonomic and functional aspects, we analyzed the proteins using BLASTP [45] and annotated the molecular functions and pathways using Uniprot [46] and KEGG [47].

To show the impact of MetaLP on the taxonomy analysis, we mapped the identified proteins at 1% FDR to the corresponding species for mock and soil datasets. For mock communities, we found that MetaLP identified significantly more proteins for the low-abundant species. Fig I in S1 Text shows the identified protein counts for the five least abundant species. Compared to the second-best protein inference tool, MetaLP was able to identify more proteins from those low-abundance organisms, which will provide more functional insights into those species. For the least abundant species (i.e., Nitrosomonas ureae), we could find more related pathways using the proteins only identified by MetaLP as shown in Table 5.

**Table 5:** Pathways found by the proteins identified only by MetaLP for the least abundant species in the mock communities.

Pathways
Alanine, aspartate and glutamate metabolism
Bacterial chemotaxis
Monobactam biosynthesis
Nucleotide excision repair
Oxidative phosphorylation
Pantothenate and CoA biosynthesis
RNA degradation
Selenocompound metabolism
Sulfur metabolism
Two-component system
Valine, leucine and isoleucine biosynthesis

**Fig 3:**
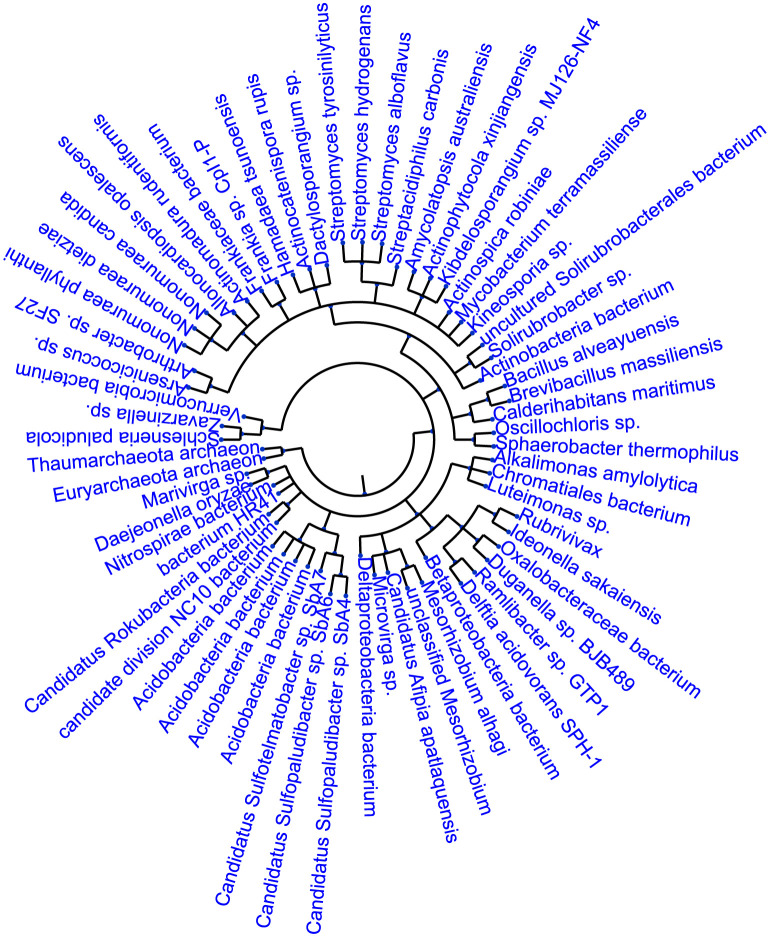
Phylogenetic tree of the species only found by the MetaLP from the soil metaproteomes.

For the soil metaproteomes, we analyzed the taxonomic profile by searching inferred proteins against the non-redundant protein database of NCBI. The phylogenetic tree for the taxa that were only detected from the inferred protein list by MetaLP is shown in Fig 3. There are 69 taxa detected by the proteins found only by MetaLP. Some taxa were found playing important roles in the soil microbial communities. For example, Vicinamibacterales is one of the major groups constituting the HM-Tol module [48], which is an indicator of metal pollution; Bradyrhizobium has positive effects on biological nitrogen fixation [49], which is beneficial for the crops; Spartobacteria is related with metabolising di-(2-ethylhexyl) phthalate (DEHP) biodegradation [50]. As shown in Table 6, there are seven more pathways using the proteins identified only by MetaLP from the least five abundant OTU clusters. Therefore, we believe that, with the information from OTU clusters, our MetaLP model can provide more sensitive protein identifications for those low-abundance species.

**Table 6:** Pathways found by the proteins identified only by MetaLP for the least abundant OTU clusters in the soil metaproteomes.

Pathways
Cysteine and methionine metabolism
Biosynthesis of various plant secondary metabolites
Metabolic pathways
Biosynthesis of secondary metabolites
Biosynthesis of cofactors
Glycerolipid metabolism
Biosynthesis of amino acids

From the functional aspect, we annotated the molecular functions for the proteins of the least abundant species (i.e., Odoribacter splanchnicus). We used proteins inferred only by MetaLP from the human gut metaproteome and the functional annotation is shown in Table 7. We found that the proteins only inferred by MetaLP are significant for drug design related to human gut and probiotics: dihydroorotate dehydrogenase inhibitors help arrest the growth of plasmodium falciparum; elongation factor G affects the adhesion to mucin, which influences the carbon source for probiotic bacteria; long-chain fatty acid plays an essential role in assembling the membrane lipids in the gut environment; fructose-bisphosphate aldolase is a crucial enzyme for gene expression of aloe polysaccharide, which may have prebiotic effects on gut microbiota; thioredoxin peroxidase is essential for Babesia microti protection against the adverse environmental factors.

**Table 7:** Molecular functions and corresponding references for the proteins of least abundant species in human gut proteome.

Protein ID	Molecular function	Reference
ADY31798	dihydroorotate dehydrogenase	[51]
ADY33332	translation elongation factor	[52]
ADY32349	long-chain fatty acid-CoA ligase	[53]
ADY31784	fructose-bisphosphate aldolase	[54]
ADY34425	thioredoxin peroxidase	[55]

### 4.5 Relation to the existing works

Supplementary biological information could be incorporated to protein inference. For example, Gerster et al. proposed a protein inference method, called MIPGEM [56]. It used a tripartite graph by including, in addition to the relationship between peptides and proteins, the connection between genes. He et al. designed a linear programming model for protein inference, called PIPQ, which viewed the protein inference problem as a special protein quantification problem [36]. These two methods and our MetaLP all used tripartite graphs to solve the protein inference problem. The difference is that MIPGEM constructed a tripartite graph of peptides, proteins, and genes, whereas MetaLP used taxonomy information not genes. In metaproteomics, a protein may be produced by more than one species. So, replacing genes by the taxa is a more general organization of proteins. Also, MIPGEM used a Markovian assumption to deal with the dependencies among peptides and proteins, but MetaLP formulated the problem as the linear optimization problem. Here, we did not compare the performance of MetaLP to MIPGEM because MIPGEM achieved comparable performance compared to ProteinProphet in its original study. PIPQ considered the protein inference problem as a protein quantification problem and the presence of one protein was determined by its abundance, whereas MetaLP used the genomic information to construct the prior of species abundance. Another linear-programming-based tool, ProteinLP [10], considered that a protein was present if at least one of its peptides was present, whereas MetaLP reported the presence of a protein by marginalizing the joint probability that the protein, its peptides, and its parent OTUs were present and measured. The results in Tables 2 and 3 demonstrate that PIPQ performed the second best in most experimental settings. The improvements made by MetaLP compared to PIPQ were correlated with the complexity of microbial communities. From Table 4, we found that PIPQ performed the second best in identifying true proteins for our synthetic dataset, but its accuracy was not as high as our MetaLP.

The benchmarked protein inference algorithms based on the bipartite graph usually assume that the proteins and their peptides are equally likely to be present and measured. However, the presence of a protein is highly related to the distribution of their parent species in the metaproteome samples. MetaLP incorporates the species abundance as the prior knowledge. Thus, the probability of a protein being present and measured is adjusted with regarding to the probabilities of its peptides and the abundances of its parent species. When there are two proteins with the same support at the peptide level, MetaLP tends to pick the one with evidence from the metagenomic sequencing data, whereas other tools may randomly pick a protein. This could be the main reason that some proteins were missed by MetaLP but reported by other benchmarked tools.

## 5 Conclusion

In this study, an integrative linear programming model, called MetaLP, was designed to generate a reliable list of proteins from identified peptides. MetaLP incorporates the taxonomic abundances as prior information and formulates the protein inference as a linear programming problem. We extracted the taxonomic abundances from the metagenomic sequencing data or the 16s rRNA gene amplicon sequencing data. The experiments on both mock and real-world microbial communities demonstrated that MetaLP obtained the highest number of protein identifications compared to five existing protein inference methods. The improvement of the metaproteomics results of microbial communities using their taxonomic information shows the value of integrated meta-omics studies.

## Supporting information

S1 Text Supplementary materials. (PDF)
