# Supplementary material for: MetaLP: An integrative linear programming method for protein inference in metaproteomics
Source: PLoS Comput Biol. 2022 Oct 21;18(10):e1010603. doi: 10.1371/journal.pcbi.1010603 (PMC9629623; doi:10.1371/journal.pcbi.1010603)
Supplement: S1 Text — (PDF) [file pcbi.1010603.s001.pdf]

Supplementary material

1 The overlap of identified proteins among the benchmarked algorithms

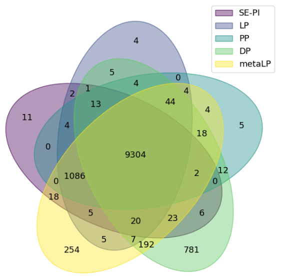

(a) Protein inference overlap for P2.

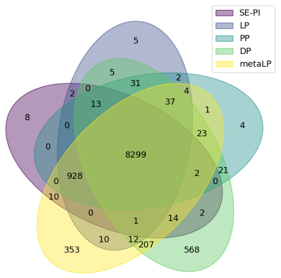

(b) Protein inference overlap for P3.

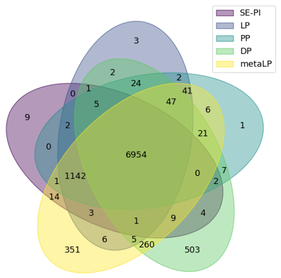

(c) Protein inference overlap for P4.

Figure A: The overlap of the inferred proteins at protein FDR 1% for mock communities

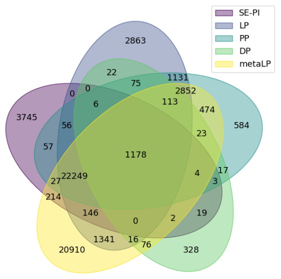

(a) Protein inference overlap for marine1.

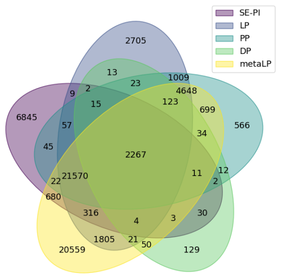

(b) Protein inference overlap for marine2.

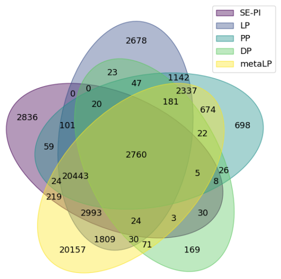

(c) Protein inference overlap for marine3.

Figure B: The overlap of the inferred proteins at protein FDR 1% for marine microbial communities

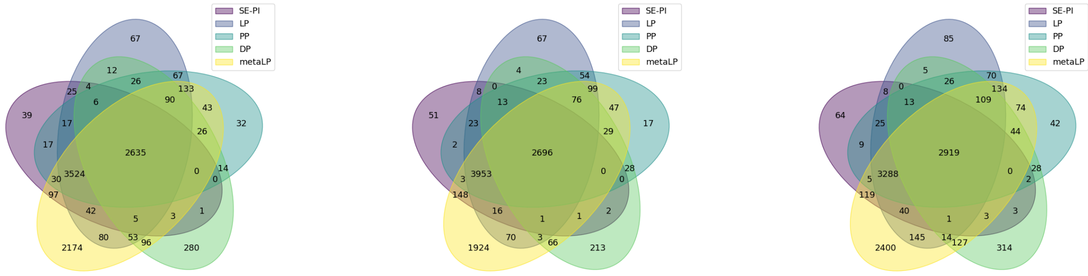

(a) Protein inference overlap for soil1. (b) Protein inference overlap for soil2. (c) Protein inference overlap for soil3.

Figure C: The overlap of the inferred proteins at protein FDR 1% for soil microbial communities

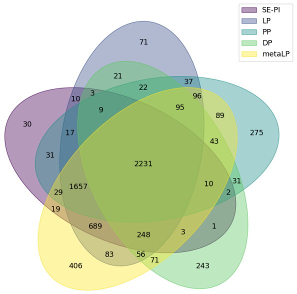

Figure D: The overlap of the inferred proteins at protein FDR 1% for the human gut microbial community

2 Impact of parameter ϵ

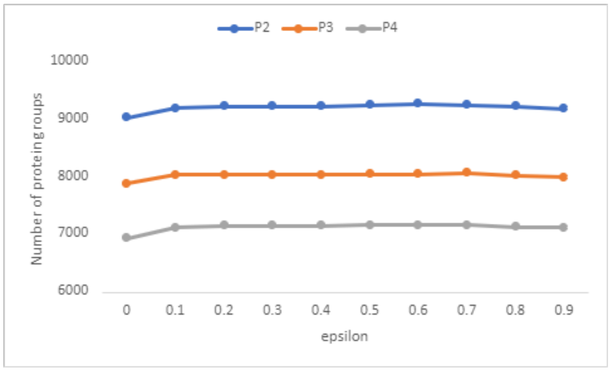

Figure E: The relationship between parameter ϵ and the number of the inferred proteins by MetaLP at protein FDR 1% on mock communities

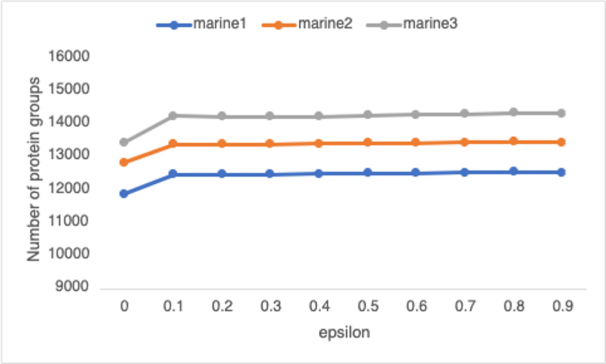

Figure F: The relationship between parameter ϵ and the number of the inferred proteins by MetaLP at protein FDR 1% on marine microbial communities

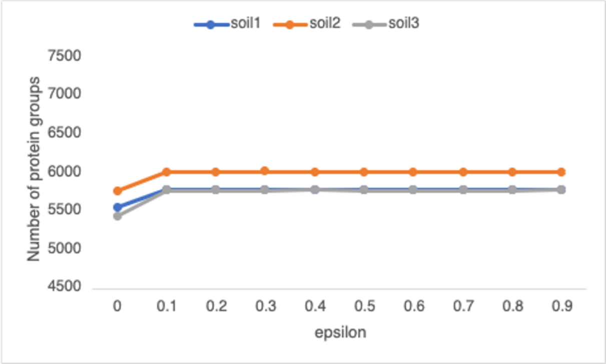

Figure G: The relationship between parameter ϵ and the number of the inferred proteins by MetaLP at protein FDR 1% on soil microbial communities

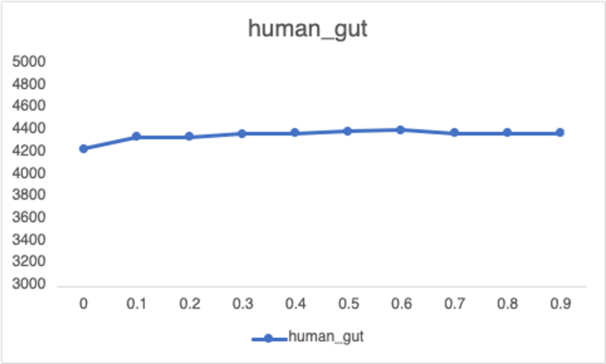

Figure H: The relationship between parameter ϵ and the number of the inferred proteins by MetaLP at protein FDR 1% on human gut microbial community

3 Inferred proteins for the less abundant species of mock communities

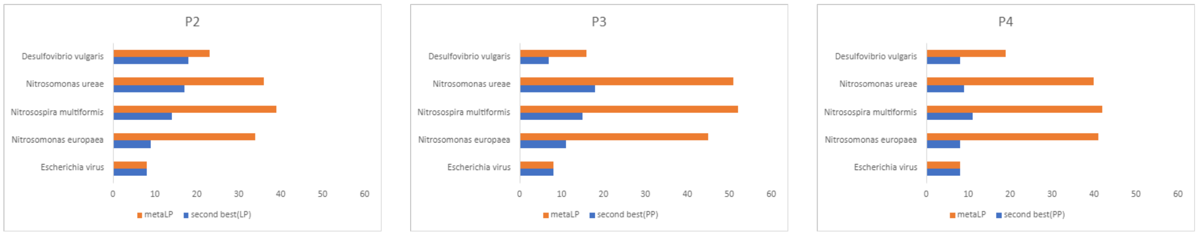

(a) P2 (b) P3 (c) P4

Figure I: The number of inferred proteins within the least abundant species for mock communities at protein FDR 1%

4 Protein inference performance using the picked target-decoy strategy

Table A: Benchmarking of protein identification at 1% FDR using three mock metaproteomes and a human gut metaproteome

	Database search engines & Filters							
	Comet with PeptideProphet				SE-SF[c]			
Metaproteomes[a] / PI tools[b]	P2	P3	P4	HG	P2	P3	P4	HG
SE-PI	8764	7631	6803	3603	9163	7238	7114	3431
ProteinLP (LP)	8784	7643	6815	3975	9289	7466	7230	3682
ProteinProphet (PP)	8793	7652	6824	3919	9274	7462	7217	3571
DeepPep (DP)	8790	7547	6752	2835	5328	2974	6912	2489
PIPQ-e	8745	7687	6809	4106	9236	6532	7003	3862
PIPQ-m	8717	7636	6859	4151	8376	7317	6528	3834
PIPQ-lp	8861	7724	6842	3894	9316	6721	7016	2931
MetaLP*	8785	7621	6801	3841	9245	7478	7207	3527
MetaLP	**9136**	**7926**	**7036**	**4244**	**9585**	**7863**	**7495**	**3945**

[a] Metaproteomes: three mock microbial communities: P2, P3, P4; a human gut metaproteome: HG
[b] Protein inference tools(PI tools): SE-PI, Sipros Ensemble protein inference; MetaLP*, MetaLP model without OTU cluster probabilities.
[c] SE-SF: Sipros-Ensemble searching and filtering
[d] The best entry was in bold and the next best was underlined.

Table B: Benchmarking of protein identification at 1% FDR using three marine metaproteomes and three soil metaproteomes

	Database search engines & Filters					
	Comet with PeptideProphet			SE-SF		
Marine Metaproteomes[a] / PI tools[b]	M1	M2	M3	M1	M2	M3
SE-PI	9122	9980	10463	11239	10947	10626
ProteinLP (LP)	10052	10902	11456	12631	11906	14040
ProteinProphet (PP)	9329	10139	10662	14209	14307	15563
DeepPep (DP)	3021	3670	3679	3433	3111	4707
PIPQ-e	8246	8483	9345	13431	10281	14411
PIPQ-m	10151	11301	10808	13010	14339	15620
PIPQ-lp	10894	11489	12020	13497	14605	15714
MetaLP*	9180	9983	10414	10939	12205	13091
MetaLP	**11880**	**12840**	**13449**	**18506**	**18374**	**18115**
Soil Metaproteomes[a] / PI tools[b]	S1	S2	S3	S1	S2	S3
SE-PI	4906	5302	4834	5927	6216	6008
ProteinLP (LP)	5021	5373	4935	6546	6474	6520
ProteinProphet (PP)	4941	5378	4946	6528	6512	6632
DeepPep (DP)	3115	3037	3451	3640	3369	4304
PIPQ-e	5289	5373	5211	5925	5632	6079
PIPQ-m	5319	5422	5231	6229	6373	6734
PIPQ-lp	5331	5559	5210	5955	5795	6667
MetaLP*	4916	5307	4849	6230	6306	6262
MetaLP	**5560**	**5769**	**5440**	**7042**	**7004**	**7544**

[a] Three marine and three soil metaproteomes M1 (Marine 1), M2 (Marine 2), M3 (Marine 3), S1 (Soil 1), S2 (Soil 2), S3 (Soil 3).
[b] Protein inference tools(PI tools): SE-PI, Sipros Ensemble protein inference; MetaLP*, MetaLP model without OTU cluster probabilities.
[c] SE-SF: Sipros-Ensemble searching and filtering
[d] The best entry was in bold and the next best was underlined.

5 Protein inference performance using all identified peptides without filtering

Table C: Benchmarking of protein identification at 1% FDR using all the identified peptides without filtering as input.

	Database search engines & Filters								
	Comet with PeptideProphet								
Metaproteomes[a] / PI tools	P2	P3	P4	M1	M2	M3	S1	S2	S3
ProteinLP (LP)	9168	7616	6710	10052	11406	11915	5123	5525	5088
ProteinProphet (PP)	9157	6964	6523	9947	10845	11208	5249	5662	5290
DeepPep (DP)	5330	6347	6010	2991	3472	3463	3339	2888	3379
MetaLP*	9137	7518	6618	9483	10342	10654	4992	5546	5073
MetaLP	**9487**	**7842**	**6862**	**12227**	**13140**	**13809**	**5362**	**6025**	**5547**

[a] Metaproteomes: three mock microbial communities: P2, P3, P4; three marine metaproteomes M1 (Marine 1), M2 (Marine 2), M3 (Marine 3); three soil metaproteomes: S1 (Soil 1), S2 (Soil 2), S3 (Soil 3).
[b] The best entry was in bold and the next best was underlined.

6 Protein inference performance at varied FDRs using the picked target-decoy strategy

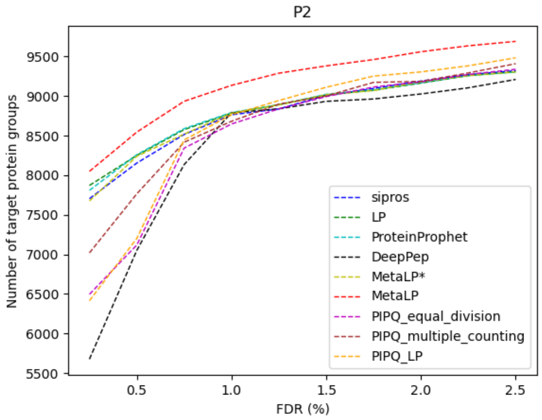

(a) P2

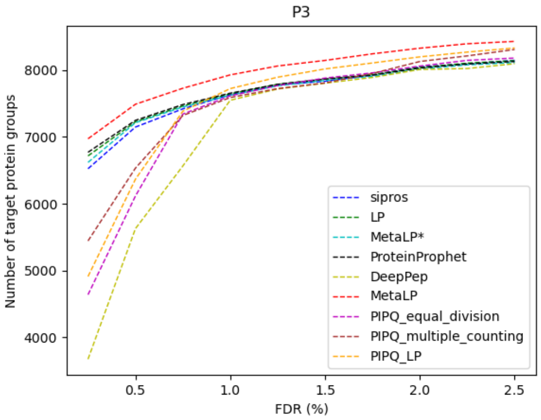

(b) P3

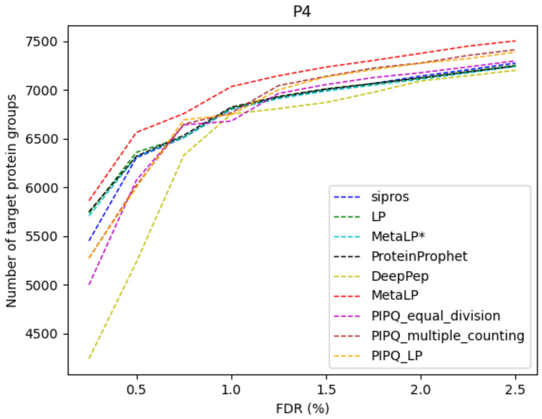

(c) P4

Figure J: The number of identified proteins at varied FDRs for mock communities

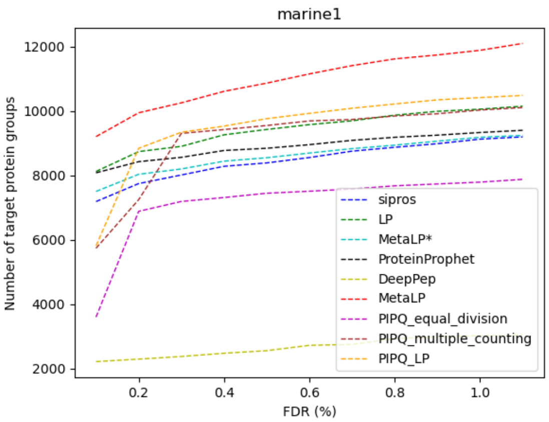

(a) marine1

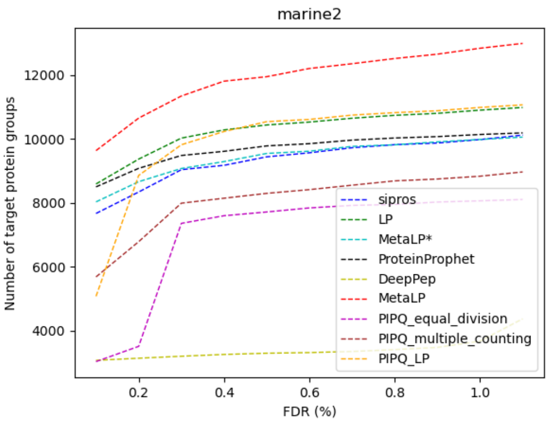

(b) marine2

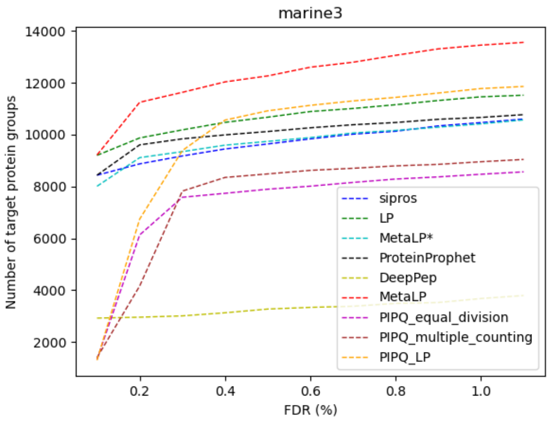

(c) marine3

Figure K: The number of identified proteins at varied FDRs for marine metaproteome data sets

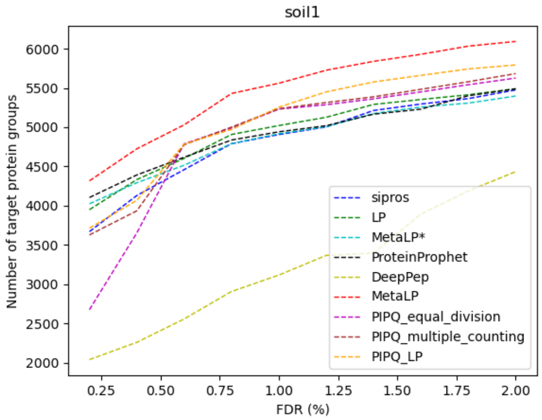

(a) soil1

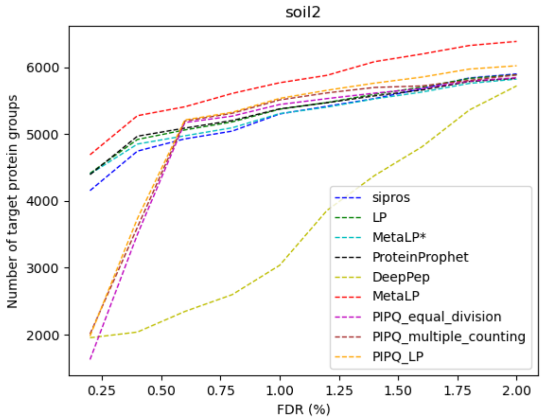

(b) soil2

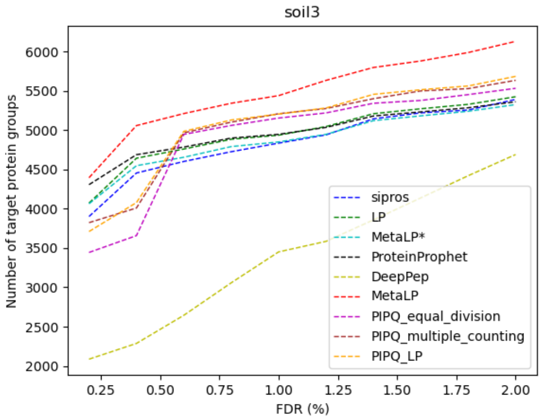

(c) soil3

Figure L: The number of identified proteins at varied FDRs for soil metaproteome data sets

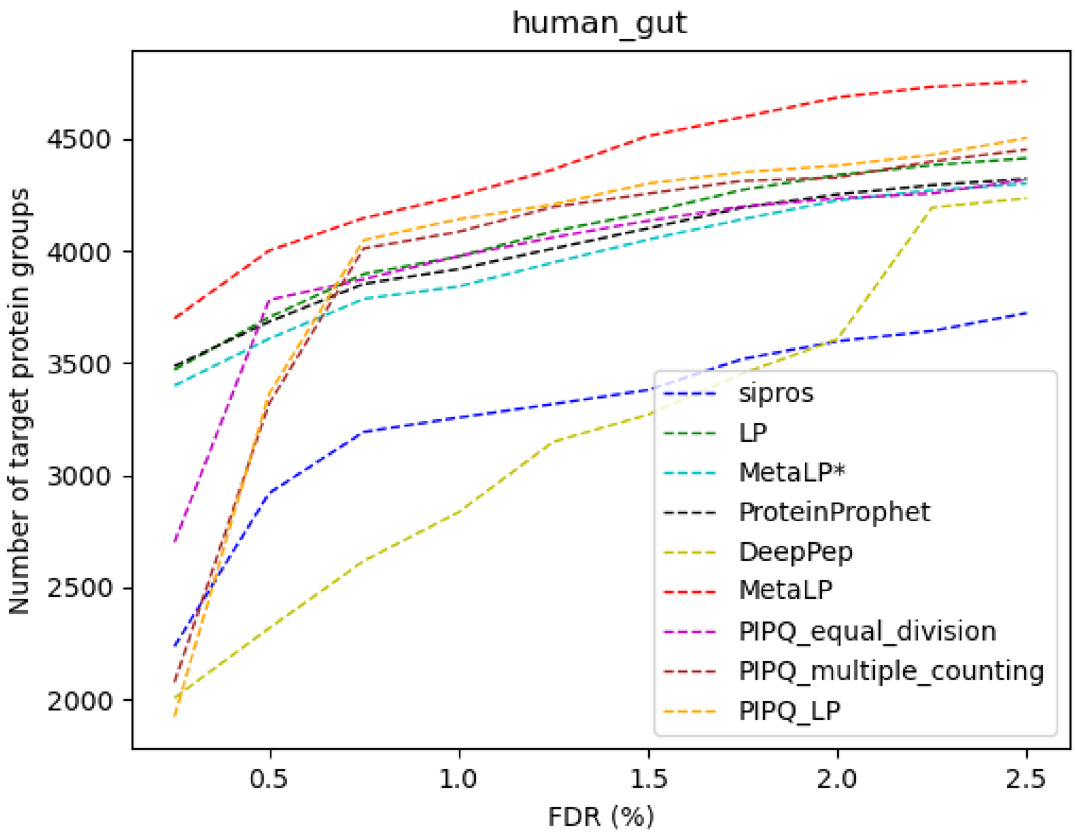

Figure M: The number of identified proteins at varied FDRs for the human gut metaproteome data set

7 Protein inference performance at varied FDRs using the target-decoy strategy

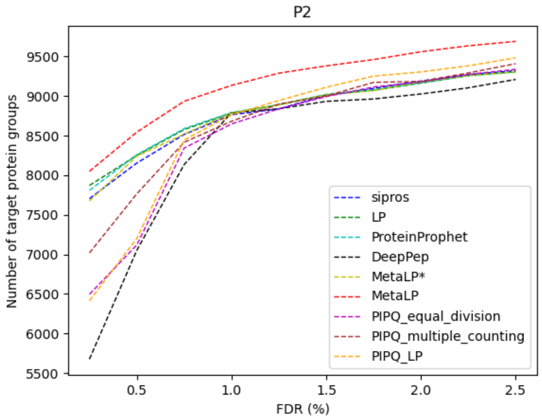

(a) P2

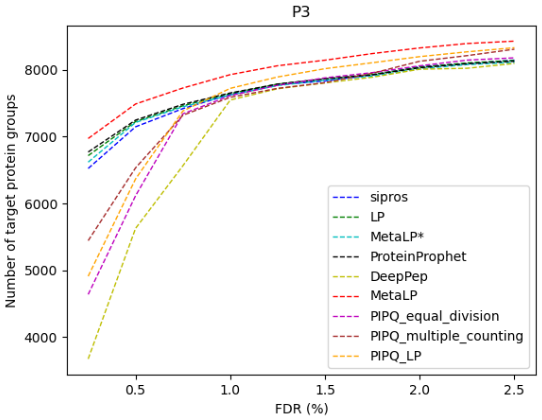

(b) P3

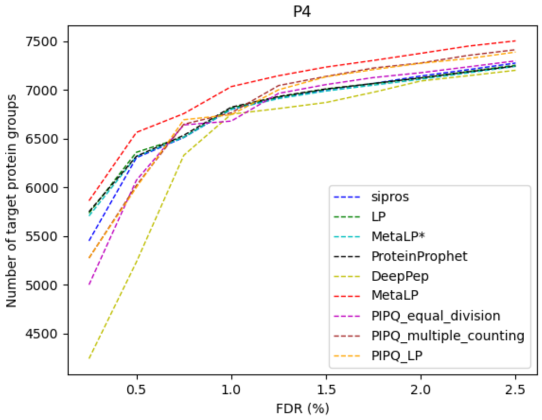

(c) P4

Figure N: The number of identified proteins at varied FDRs for mock communities

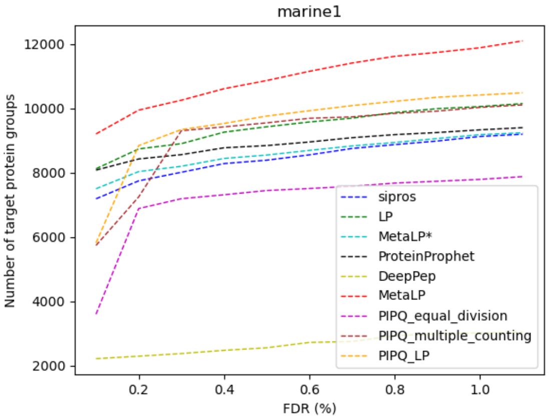

(a) marine1

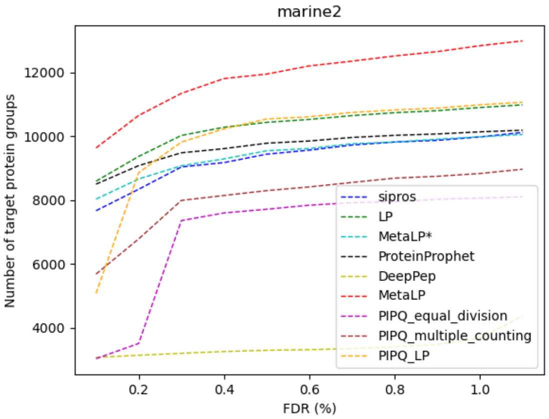

(b) marine2

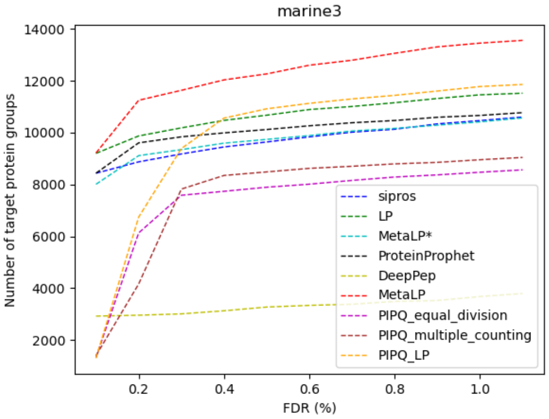

(c) marine3

Figure O: The number of identified proteins at varied FDRs for marine metaproteome data sets

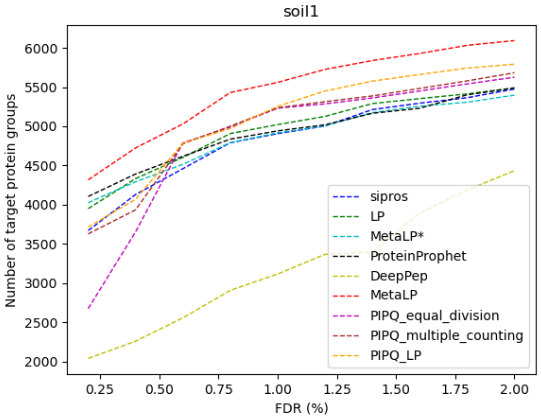

(a) soil1

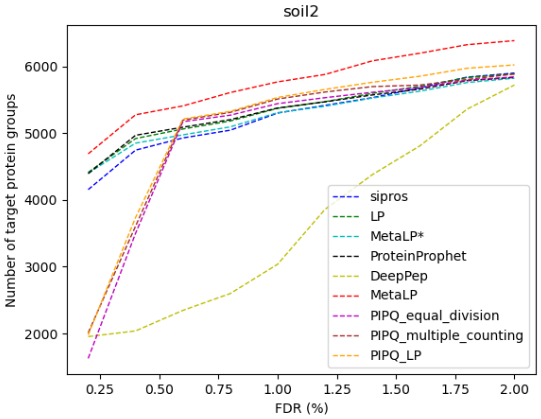

(b) soil2

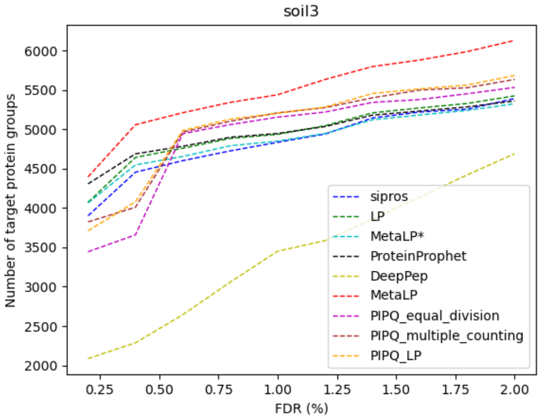

(c) soil3

Figure P: The number of identified proteins at varied FDRs for soil metaproteome data sets

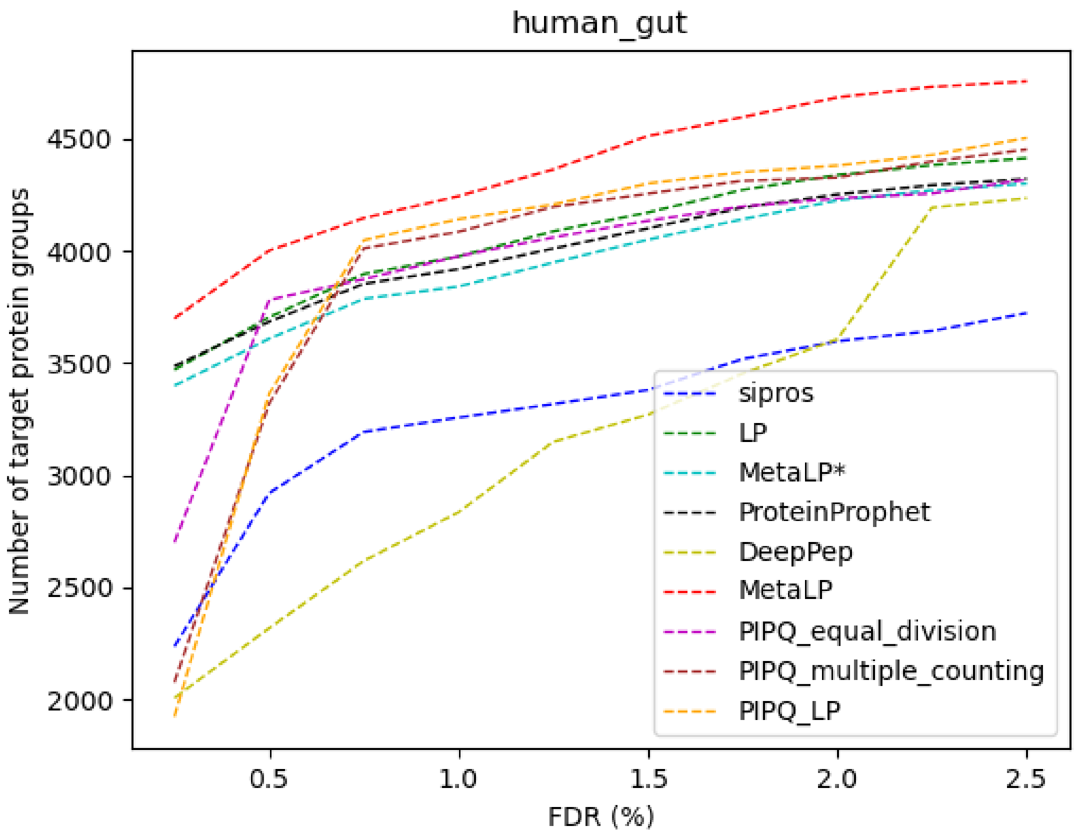

Figure Q: The number of identified proteins at varied FDRs for the human gut metaproteome data set

8 Time and memory usage

Table D: Time and memory usage

	P2	M1	S1	HG
#peptides	13,043	25,411	11,727	8,585
#proteins	35,890	123,538	16,739	15,560
time	3.34 s	32.38 s	3.22 s	2.03 s
RAM	0.3 GB	2.5 GB	0.2 GB	0.2 GB

9 Accuracy estimation

Table E: Accuracy on proteins containing degenerate peptides

	PP[a]		LP		DP		MetaLP*		MetaLP		PIPQ-e		PIPQ-m		PIPQ-lp	
	TP	FP	TP	FP	TP	FP	TP	FP	TP	FP	TP	FP	TP	FP	TP	FP
P2 (k=5000)																
Simple[b]	2423	0	2349	0	2540	9	2359	0	2332	0	2508	6	2581	4	2474	6
Degenerate[c]	2577	0	2651	0	2450	1	2641	0	2668	0	2485	1	2414	1	2519	1
P3 (k=5000)																
Simple	2412	2	2487	1	2679	18	2445	3	2396	3	2516	16	2463	10	2442	11
Degenerate	2585	1	2511	1	2303	0	2551	1	2600	1	2467	1	2526	1	2545	1
P4 (k=5000)																
Simple	2616	6	2649	6	2891	20	2623	6	2540	4	2643	12	2463	8	2471	12
Degenerate	2378	0	2345	0	2088	1	2370	1	2455	1	2337	0	2529	0	2517	0
Marine1 (k=8000)																
Simple	1897	7	1878	7	2568	184	2069	12	1655	3	2825	125	1888	5	1842	10
Degenerate	6094	1	6114	1	5229	19	5917	2	6340	2	5044	6	6105	2	6144	4
Marine2 (k=8000)																
Simple	1935	4	1969	2	2811	170	1969	2	1695	3	2469	59	2091	6	1801	11
Degenerate	6061	0	6029	0	5002	17	6029	0	6302	0	5468	4	5901	2	6183	5
Marine3 (k=8000)																
Simple	1743	4	1867	5	2535	181	1978	7	1631	4	2044	22	1945	5	1776	17
Degenerate	6250	3	6127	1	5270	14	6012	3	6362	3	5931	3	6048	2	6198	9
Soil1 (k=4500)																
Simple	2130	22	2129	24	2477	60	2176	26	1978	14	2098	28	2057	22	1971	28
Degenerate	2347	1	2346	1	1960	3	2297	1	2507	1	2374	0	2421	0	2499	2
Soil2 (k=4500)																
Simple	2276	12	2359	12	2477	60	2388	12	2213	8	2341	20	2329	14	2296	20
Degenerate	2212	0	2129	0	1960	3	2100	0	2279	0	2138	1	2155	2	2183	1
Soil3 (k=4500)																
Simple	2017	11	2068	12	2194	80	2111	15	1905	9	2030	22	2001	11	2025	22
Degenerate	2472	0	2419	1	2221	5	2372	2	2585	1	2448	0	2487	1	2453	0
Human gut (k=3000)																
Simple	1466	0	1557	2	1600	117	1553	1	1455	0	1592	6	1581	3	1517	6
Degenerate	1531	3	1438	3	1273	0	1443	3	1542	3	1401	1	1411	5	1471	6

[a] Protein inference algorithms: PP, ProteinProphet; LP, ProteinLP; DP, DeepPep; MetaLP*, MetaLP model without OTU cluster probabilities.
[b] Simple: 'simple proteins', contain at least one unique peptide not shared by any other proteins.
[c] Degenerate: 'degenerate proteins' share peptides with other proteins

10 Protein inference performance of MetaLP using the sequencing depth to estimate the OTU probability

We used the sequencing depth to estimate the OTU probability. The probability of the OTU clusters was calculated as in Eq. A. The contigs were assembled from DNA sequencing reads. We denote the MetaLP using the sequencing depth as MetaLP$^+$ and applied it to the mock, marine, and soil microbial samples. Since human gut data provided 16s rRNA sequencing data, it is not necessary to consider the sequencing depth. The Benchmarking of protein identification at 1% FDR were shown in Tables F and G. MetaLP$^+$ achieved the best performance compared to the other tools, including two other variants of MetaLP.

$$P(otu_k) = \frac{\sum_{Read_i \in OTU\ Cluster_k} |Read_i|}{\sum_{Contig_j \in OTU\ Cluster_k} |Contig_j|} \tag{A}$$

Table F: Benchmarking of protein identification at 1% FDR using three mock metaproteomes and a human gut metaproteome

	Database search engines & Filters							
	Comet with PeptideProphet				SE-SF[c]			
Metaproteomes[a] / PI tools[b]	P2	P3	P4	HG	P2	P3	P4	HG
SE-PI	8627	7524	6605	3602	9153	7258	7006	3393
LP	8684	7605	6686	3945	9168	7324	7038	3682
PP	8678	7620	6579	3895	9157	7327	7010	3571
DP	8641	7617	6452	2762	5330	2758	6916	2370
PIPQ-e[d]	8648	7612	6681	3976	9193	6403	6926	3872
PIPQ-m[d]	8682	7587	6766	4085	8158	7201	6463	3843
PIPQ-lp[d]	8764	7721	6746	4141	9206	6657	6933	2931
MetaLP*	8663	7581	6676	3832	9137	7277	7007	3527
MetaLP	9032	7883	6937	4233	9487	7669	7335	4004
MetaLP$^+$	**9046**	**7890**	**6947**	**–**	**9490**	**7689**	**7349**	**–**

[a] Metaproteomes: three mock microbial communities: P2, P3, P4; a human gut metaproteome: HG.
[b] Protein inference tools(PI tools): SE-PI, Sipros Ensemble protein inference; LP, ProteinLP; PP, ProteinProphet; DP, DeepPep; MetaLP*, MetaLP model without OTU cluster probabilities.
[c] SE-SF: Sipros-Ensemble searching and filtering.
[d] PIPQ with three different options, i.e., PIPQ-e (equal division), PIPQ-m (multiple counting), PIPQ-lp (linear programming).
[e] The best entry was in bold, the second best was underlined.

Table G: Benchmarking of protein identification at 1% FDR using three marine metaproteomes and three soil metaproteomes

	Database search engines & Filters					
	Comet with PeptideProphet			SE-SF		
Marine Metaproteomes[a] / PI tools[b]	M1	M2	M3	M1	M2	M3
SE-PI	9183	9990	10465	11160	10720	10405
LP	10052	10903	11442	15021	14290	14042
PP	9329	10127	10655	14162	14221	15547
DP	3021	3608	3679	3435	2758	4657
PIPQ-e[d]	7789	8063	8471	14052	10035	15249
PIPQ-m[d]	10027	8833	8954	12894	14136	16470
PIPQ-lp[d]	<u>10412</u>	<u>10986</u>	<u>11775</u>	<u>15532</u>	<u>15441</u>	<u>16619</u>
MetaLP*	9180	9964	10412	13387	12248	11062
MetaLP	11881	12826	13342	18562	18228	18030
MetaLP$^+$	**12025**	**12966**	**13612**	**18671**	**18435**	**18204**
Soil Metaproteomes[a] / PI tools[b]	S1	S2	S3	S1	S2	S3
SE-PI	4901	5272	4906	5859	6216	6005
LP	5021	5373	4935	<u>6546</u>	6464	6520
PP	4941	5378	4946	6528	<u>6512</u>	6632
DP	3115	3037	3427	3640	3369	4307
PIPQ-e[d]	5231	5443	5153	5905	5553	6079
PIPQ-m[d]	5236	5512	<u>5208</u>	6229	6339	<u>6739</u>
PIPQ-lp[d]	<u>5255</u>	<u>5535</u>	5203	5955	5748	6662
MetaLP*	4916	5303	4849	6230	6306	6262
MetaLP	5516	5769	5440	7042	7013	7544
MetaLP$^+$	**5658**	**5942**	**5669**	**7068**	**7210**	**7686**

[a] Three marine and three soil metaproteomes M1 (Marine 1), M2 (Marine 2), M3 (Marine 3), S1 (Soil 1), S2 (Soil 2), S3 (Soil 3).
[b] Protein inference tools(PI tools): SE-PI, Sipros Ensemble protein inference; LP, ProteinLP; PP, ProteinProphet; DP, DeepPep; MetaLP*, MetaLP model without OTU cluster probabilities.
[c] SE-SF: Sipros-Ensemble searching and filtering
[d] PIPQ with three different options, i.e., PIPQ-e (equal division), PIPQ-m (multiple counting), PIPQ-lp (linear programming).
[e] The best entry was in bold, the second best was underlined.
